# Toward a statistical validation of brain signatures as robust measures of behavioral substrates

**DOI:** 10.1002/hbm.26265

**Published:** 2023-03-20

**Authors:** Evan Fletcher, Sarah Farias, Charles DeCarli, Brandon Gavett, Keith Widaman, Fransia De Leon, Dan Mungas

**Affiliations:** ^1^ Department of Neurology University of California, Davis Davis California USA; ^2^ School of Psychological Science University of Western Australia Perth Australia; ^3^ School of Education University of California, Riverside Riverside California USA; ^4^ School of Medicine University of California, Davis Davis California USA

**Keywords:** behavior domains, brain signatures, statistical validation

## Abstract

The “brain signature of cognition” concept has garnered interest as a data‐driven, exploratory approach to better understand key brain regions involved in specific cognitive functions, with the potential to maximally characterize brain substrates of behavioral outcomes. Previously we presented a method for computing signatures of episodic memory. However, to be a robust brain measure, the signature approach requires a rigorous validation of model performance across a variety of cohorts. Here we report validation results and provide an example of extending it to a second behavioral domain. In each of two discovery data cohorts, we derived regional brain gray matter thickness associations for two domains: neuropsychological and everyday cognition memory. We computed regional association to outcome in 40 randomly selected discovery subsets of size 400 in each cohort. We generated spatial overlap frequency maps and defined high‐frequency regions as “consensus” signature masks. Using separate validation datasets, we evaluated replicability of cohort‐based consensus model fits and explanatory power by comparing signature model fits with each other and with competing theory‐based models. Spatial replications produced convergent consensus signature regions. Consensus signature model fits were highly correlated in 50 random subsets of each validation cohort, indicating high replicability. In comparisons over each full cohort, signature models outperformed other models. In this validation study, we produced signature models that replicated model fits to outcome and outperformed other commonly used measures. Signatures in two memory domains suggested strongly shared brain substrates. Robust brain signatures may therefore be achievable, yielding reliable and useful measures for modeling substrates of behavioral domains.

## Graphical abstract

We implement a method for computing data‐driven signatures of behavior outcomes that are robust across validation cohorts. This suggests the method may be used to develop robust brain phenotypes in brainwise association studies.

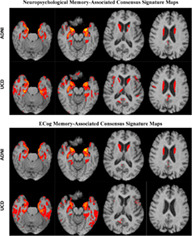

## 1 INTRODUCTION

The “brain signature of cognition” concept has garnered interest as a data‐driven, exploratory approach to better understanding key brain regions involved in specific cognitive functions, with the potential to maximally account for brain substrates of behavioral outcomes. It has been characterized as discovering “statistical regions of interest” (sROIs or statROIs) (Chen et al., 2010; Fletcher et al., 2013; Hua et al., 2009) or brain “signature regions” associated with outcomes (Arenaza‐Urquijo et al., 2019; Dickerson et al., 2009; Fletcher, Gavett, et al., 2021; Gross et al., 2012). For a variable of interest (in our study, gray matter [GM] thickness), it computes areas of the brain that are most associated to a behavior outcome of interest. To be a robust brain measure, a signature requires validation, showing model fit to outcome replicability in multiple datasets beyond the discovery set where it was developed. If signatures are separately developed in two discovery cohorts, they should also show consistent spatial selection of the signature regions. These key properties are summarized as model fit and spatial extent replicability.

The signature approach represents an evolution from theory‐driven or lesion‐driven approaches that were feasible using smaller datasets and lower computational power. Although those approaches yielded many valuable insights into brain–behavior associations, they may have missed subtler but significant effects, thus giving incomplete accounts of brain substrates of an outcome of interest. In recent years, high‐quality brain parcellation atlases have enabled a more exploratory approach, seeking combinations of atlas regions of interest (ROIs) that best associate to behaviors of interest. A shortcoming of all approaches using predefined ROIs, however, is that brain‐behavior associations may cross ROI boundaries, recruiting subsets of multiple regions but not using the entirety of a region. This may mean that a combination of atlas ROIs cannot optimally fit an outcome of interest (Jolly & Hampshire, 2021).

The signature approach aims to address these limitations. It selects features associated to outcome in a data‐driven manner. When implemented at a fine‐grained (e.g., voxel) level of feature selection, it does not need predefined ROIs. The approach we use here is direct computation of voxel‐based regressions. However, other recent implementations of exploratory feature selection have used machine learning algorithms such as support vector machines (Fan et al., 2005), support vector classification (Marek et al., 2022), relevant vector regression (Caballero et al., 2016), and deep learning using convolutional neural nets (Dinsdale et al., 2020). Machine learning may be especially promising when investigating complex multimodal brain associations with behavioral or clinical outcomes (Lee et al., 2019). Their challenge, however, is interpretability of the results, since machine learning models can be like a black box (Bach et al., 2015). This is starting to be addressed (Böhle et al., 2019). In any case, these all represent alternative implementations of the data‐driven approach.

Because it is based on data‐driven exploration, the signature approach has the potential to provide as complete an accounting of brain‐behavior associations as current technology will allow. However, approaching this ideal could require large data sets (Marek et al., 2022; Masouleh et al., 2019) that are only recently becoming available (e.g., U.K. Biobank, Sudlow et al., 2015). Both studies found that replicability depended on discovery in large dataset sizes, with (Marek et al., 2022) finding that sizes in the thousands were needed. Pitfalls of using too‐small discovery sets include inflated strengths of associations and loss of reproducibility (Marek et al., 2022). Masouleh et al. also found that replicability of model fit and consistent spatial selection depended on cohort heterogeneity including a full range of variability in brain pathology and cognitive function, the outcome domain of interest, and size of discovery set.

An algorithm that can meet these challenges by generating reproducible brain signatures is thus a worthwhile goal. In our recent work (Fletcher, Gavett, et al., 2021), we described a method for computing brain GM signatures of episodic memory in cognitively diverse populations and validated it across three independent cohorts. We found promising support for fit and spatial reproducibility. However, in‐discovery‐set versus out‐of‐set performance bias was still evident, and we did not investigate whether signature models generated in different cohorts would perform comparably across many different validation sets. Since then, following (Masouleh et al., 2019), we hypothesized that by implementing the discovery phase of our earlier algorithm in parallel across many randomly selected subsets and then aggregating, we could overcome the pitfalls and produce a reproducible and useful brain signature phenotype.

The present study therefore has two aims. The first is to rigorously test the replicability and explanatory properties of the method in our previous effort, now augmented to leverage multiple discovery set generation and aggregation. The second is to extend the method to another behavior domain: everyday memory function, measured by the Everyday Cognition scales (ECog), an informant‐based scale for measuring subtle changes in day‐to‐day function of older participants (Farias et al., 2008). We hypothesized that this could serve to illustrate the usefulness of validated signatures for discerning and comparing brain substrates of different behavioral domains.

## 2 MATERIALS AND METHODS

### 2.1 Imaging cohorts

We used discovery and validation sets drawn from two imaging cohorts. For discovery, we used 578 participants from the UC Davis (UCD) Alzheimer's Disease Research Center Longitudinal Diversity Cohort and 831 participants from the Alzheimer's Disease Neuroimaging Initiative Phase 3 cohort (identified in the following as ADNI 3), downloaded from the ADNI site (adni.loni.usc.edu). All subjects had neuropsychological and everyday function (ECog) evaluations and one MRI scan taken near the time of evaluation. For validation, we used cohorts consisting of an additional 348 participants drawn from UCD and 435 participants from ADNI Phase 1 (ADNI 1). All UCD participants had both ECog and neuropsychological measures, but in ADNI 1, consisting of an earlier series, the ECog data were not complete. The validation cohorts were separate from the discovery cohorts.

One of the aims of the UCD ADRC cohort is to explore heterogeneity of cognitive trajectories in aging associated with a mixture of pathologies among an ethno‐racially diverse group of older adults.

The ADNI project was launched as a public–private partnership in 2003 by the National Institutes of Aging, the National Institute of Biomedical Imaging and Bioengineering, the Food and Drug Administration, private pharmaceutical companies, and nonprofit organizations. The primary goal of ADNI is to test whether serial MRI, PET, other biomarkers, and clinical and neuropathological assessment can be combined to measure progression of MCI and early Alzheimer's disease (AD). The principal investigator is Michael Weiner, MD, VA Medical Center and University of California, San Francisco. For current information on ADNI, see www.adni-info.org.

### 2.2 Cognitive and everyday function assessment

Cognitive assessments of episodic memory were based on the Spanish and English Neuropsychological Assessment Scales (SENAS) (Mungas et al., 2004; Mungas, Reed, Tomaszewski Farias, & DeCarli, 2005) within the UCD ADRC cohort. SENAS is a composite measure based on a 15 item verbal list learning test incorporating performance across five learning trials and immediate recall. The memory composite from the ADNI cohort (ADNI‐Mem) (Crane et al., 2012) was based on similar items from a list learning test as well as memory items from the Alzheimer's Disease Assessment Scale‐Cognitive Subscale (ADAS‐Cog) and the Mini‐Mental State Examination (MMSE). Both are sensitive to individual differences across the full range of episodic memory performance. The Everyday Memory domain from the ECog (ECogMem) (Farias et al., 2008; Farias et al., 2013) was used to measure everyday memory for both cohorts. The ECog is an informant‐rated measure of several domains relevant to cognition as it applies to daily function. It was designed to address functional abilities of older adults, particularly focusing on subtle changes in everyday function spanning preclinical AD to moderate dementia (Farias et al., 2008).

### 2.3 MRI image processing

We used single MRI scans in each cohort from the UCD and ADNI 3 cohorts.

Whole head structural T1 MRI images were processed by in‐house pipelines developed in our laboratory and described previously (Fletcher et al., 2014). The first pipeline step produced brain extractions based on convolutional neural net recognition of intracranial cavity followed by human quality control (Fletcher, Decarli, et al., 2021). This was followed by affine and B‐spline registration (Rueckert et al., 2006) of the intracranial cavity image to a structural template image, then native‐space tissue segmentation into gray (GM) white (WM) and CSF (Fletcher et al., 2012) and white matter hyperintensities with the aid of each subject's coregistered native T1 and FLAIR images (Decarli et al., 2013). Our template was constructed in‐house as a minimal deformation age‐appropriate template (Kochunov et al., 2002) with voxel sizes 0.977, 1.5, and 0.977 in the *x*, *y*, and *z* directions.

### 2.4 Gray matter density quantification

We quantified brain cortical GM by GM density measures, performed at the voxel level in each native space image using the DiReCT diffeomorphic algorithm (Das et al., 2009) applied to the segmented GM. DiReCT is a “volume‐based” or 3D algorithm (i.e., it assigns a density measure to each GM voxel) as opposed to the method employed in the commonly used Freesurfer package, which is “surface‐based” (calculating vertex‐wise distances between inner and outer 2D GM surface meshes) (Fischl & Dale, 2000). We used a voxel‐based measure because this is required by our method and translating from vertex to voxel values would be cumbersome and imprecise. There have also been some reports that the volume‐based methods are superior in some prediction situations (Schwarz et al., 2016; Tustison et al., 2014). Resulting native‐space GM density maps were deformed to template space using the affine and B‐spline parameters previously computed in our pipeline.

### 2.5 Signature variable analyses

**FIGURE 1:**
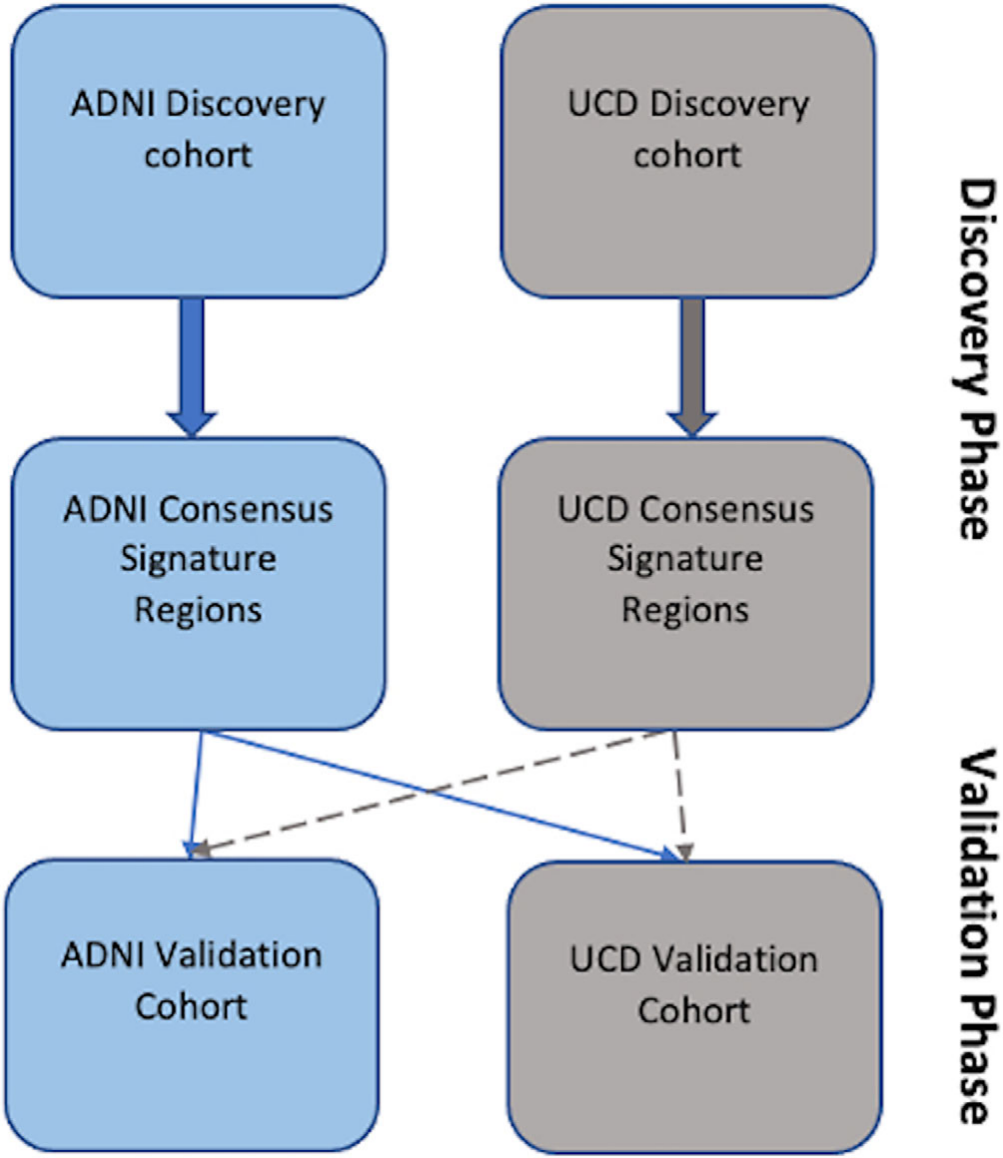
Discovery and validation of signature models. Top‐level schematic of discovery‐validation steps for both models. Signature models undergo parallel discovery steps followed by cross‐validation in two independent validation cohorts.

This paragraph gives a high‐level outline of our process (Figure 1). We computed signature models in each discovery cohort. We first generated signature masks of GM thickness association to outcome in each of 40 randomly selected discovery sets (*N* = 400 for each discovery set) within each cohort (top row in Figure 1). Separate masks were generated at each of three levels of association using regression *β* coefficient *t*‐value thresholds (*t* = 3, 5, 7). Next, for each of the *t* levels, we combined all 40 signature masks into cohort‐specific overlap frequency maps. From frequency maps, we selected cohort “consensus” masks consisting of voxels contained within at least 70% of the 40 signature masks (middle row of Figure 1). For convenience, we designate the consensus masks as TsROIs (for *t*‐level *s*ignature *ROI*) in the sequel. The 70% threshold was motivated by the maximal frequencies of locations selected in the previous report (Masouleh et al., 2019). In the validation steps, we tested cohort consensus signature models by comparing their performances with each other in each of 50 random subsets of each validation cohort (bottom row of Figure 1). Finally, we also compared consensus signature models with other, competing models of outcome in the full UCD and ADNI validation cohorts.

#### 2.5.1 Discovery of consensus models

**FIGURE 2:**
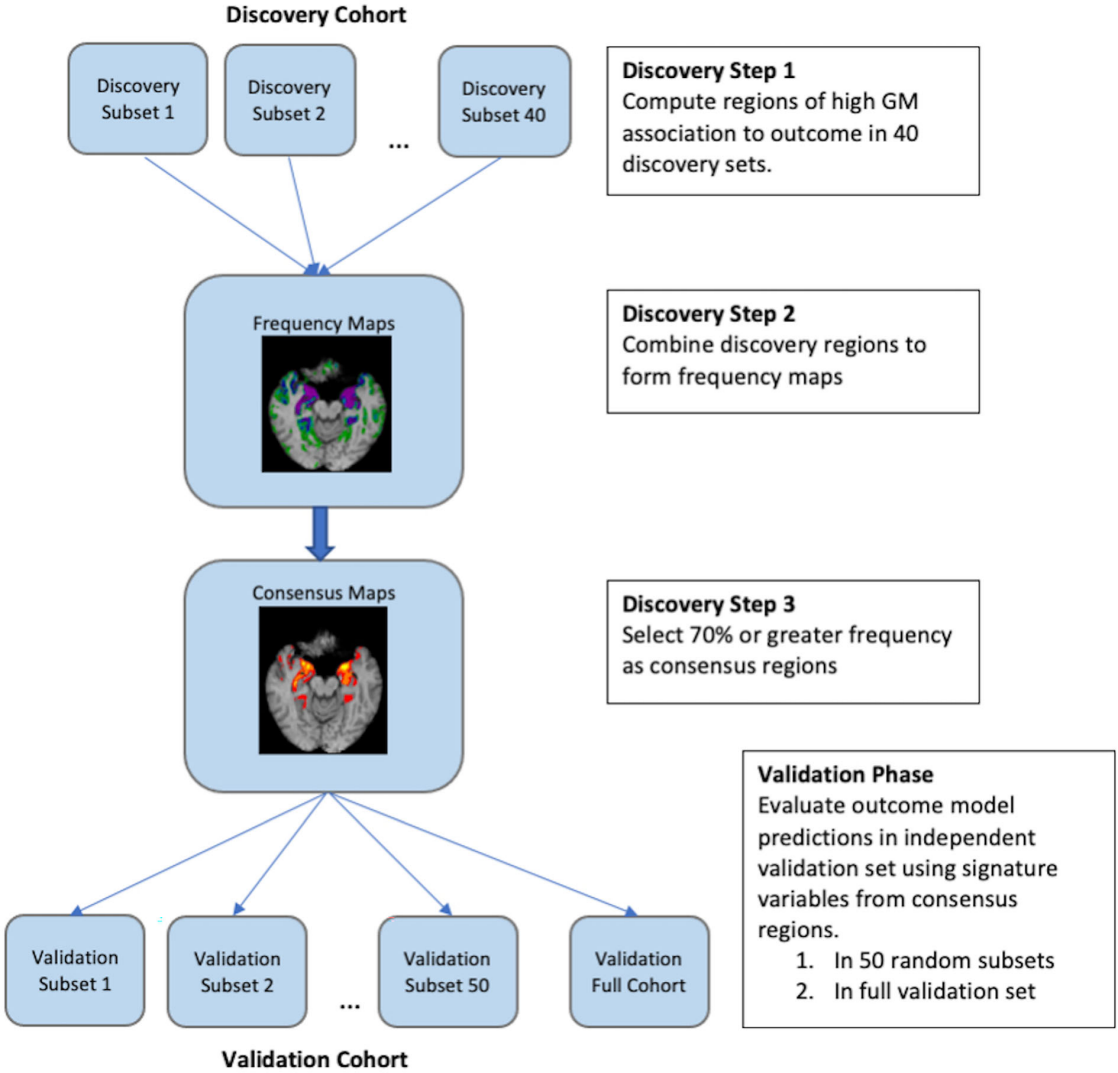
Detail of discovery and validation steps. Breakdown of analyses across randomly chosen subsets for both discovery and validation, and for validation, also including the full cohort.

Consensus models were based on discovery steps followed by aggregation. Here we provide a detailed description of this method, following Figure 2.

#### 2.5.2 Discovery step 1. Leveraging random subsets to generate variability

This step is summarized in Figure 2, top row. To augment the limited variability in a discovery cohort that is smaller than several thousand as recently recommended (Marek et al., 2022), we used 40 randomly generated subsets of size 400, consistent with size recommendations in the earlier use of this method (Masouleh et al., 2019). These subsets had pairwise nonzero overlaps, larger in UCD than in ADNI because our UCD cohort was smaller, but they also provided quasi‐independent, parallel learning of regional brain masks associated to outcome in each discovery subset.

Mask generation was extensively described in our previous work (Fletcher, Gavett, et al., 2021) and will be briefly summarized here. This had two steps. First, we generated *t*‐maps of association to outcome. Regressions were performed at each template‐space voxel with outcome domain as the dependent variable, GM density as the independent variable of interest, and controlling for age, gender, and education. The resulting GM maps of voxel‐based *t*‐values (i.e., the *t*‐value of regression *β* coefficient for GM density) indexed the strength of association of GM at every voxel. Second, we aggregated the *t*‐maps into clusters of significant association corresponding to a *t*‐value threshold. We performed nonparametric *t*‐threshold cluster size computations (Nichols & Holmes, 2001) using 2000 iterations separately for *t*‐thresholds of 3, 5, and 7. This computed an empirical distribution of cluster sizes under the null hypothesis of no association between brain and behavior outcome. Clusters from the original regressions with size in the top 5% (95th percentile) of this distribution were retained as significant. In practice, most regions of interest selected for signature masks were in the highest 0.05% (i.e., they were the largest clusters over the 2000 repetitions). Each discovery set thus produced three significant clusters, corresponding to the three *t*‐value thresholds, for each outcome domain. These were the signature masks for a discovery set, TsROI_i_ for *i* = 1, 2, 3, corresponding to *t*‐values of 3, 5, 7.

#### 2.5.3 Discovery steps 2 and 3. Frequency maps and consensus signatures

These steps are shown in Figure 2, Frequency and Consensus maps boxes. We computed overlap frequency maps of TsROI masks from all 40 discovery subsets. We then defined our consensus signature masks at each level of *t* = 3, 5, 7 as the set of voxels in template GM that were contained in at least 70% of the 40 TsROI masks at the given *t* value. Consensus signature models for an outcome then consisted of GM means in each of the three consensus TsROI masks for each participant in the target set.

For the tests we conducted, it was convenient to use regressions involving a single “signature variable” S. S was the set of predicted values from the regression

(1) $$Y=\beta_{0}+\beta_{1}\;{\text{TsROI}}_{1}+\beta_{2}\;{\text{TsROI}}_{2}+\beta_{3}\;{\text{TsROI}}_{3}$$

in a target set of interest. Here, *Y* is an outcome of interest (one of the domains considered in this paper, neuropsychological or ECog memory), and the TsROI variables represent mean values for GM thickness in each TsROI mask (*i* = 1, 2, 3 corresponding to *t*‐value thresholds of 3, 5, 7).

#### 2.5.4 Validating signature models

Validation was performed for each signature model in two separate validation sets. This corresponds to the bottom row of Figure 2.Validation sets do not overlap discovery sets. Validation first compared performance of the UCD and ADNI signature models across randomly selected subsets of each validation cohort. Then we evaluated the two signature models compared to other commonly used models of outcome in each of the entire validation cohorts.

#### 2.5.5 Testing replicability for two signature models

In each of 50 validation subsets from a validation cohort, we computed the fit of each consensus signature model to outcome, controlling for age, gender, and education (Equation 1). Overall fit was measured by adjusted R^2^.

(2) $$Y=\beta_{0}+\beta_{1}\;S+\beta_{2}\;\mathrm{age}+\beta_{3}\;\text{gender}+\beta_{4}\;\text{education}$$

For comparison, we also computed explanation of variance by demographics alone:

(3) $$Y=\beta_{0}+\beta_{1}\;\mathrm{age}+\beta_{2}\;\text{gender}+\beta_{3}\;\text{education}$$

#### 2.5.6 Testing optimal performance of signature models

We compared the fit performances of the consensus signatures against those of other brain variables within each of the entire ADNI and UCD validation cohorts. From a GM cortical parcellation atlas (https://mindboggle.info/), augmented by in‐house delineations of hippocampus, amygdala and caudate, we selected four regions most heavily overlapped by each of the consensus masks in at least one cohort. The Mindboggle Atlas (Klein et al., 2017; Klein & Tourville, 2012) is a current and commonly accepted update of the Desikan‐Killiany‐Tourville cortical parcellation scheme (Desikan et al., 2006) used in Freesurfer (Gross et al., 2012). The Mindboggle regions are defined on the ICBM‐152 template (https://www.bic.mni.mcgill.ca/ServicesAtlases/ICBM152NLin2009) and were transformed to our in‐house template using a nonlinear deformation followed by a voting scheme to resolve boundary ambiguities. The regions we selected were the amygdala, entorhinal cortex, hippocampus, and caudate. We regressed an outcome on each of these variables in turn, controlling for age, gender, and education, and tabulated the adjusted *R* ^2^ fit measures. In addition to these single‐ROI models, we made a multivariate model incorporating all these ROIs model as predictors (the “FourROIs” model).

#### 2.5.7 Evaluating significant differences in model fit

We estimated confidence intervals of adjusted *R* ^2^ fit differences between signature models and fits of the two highest fitting nonsignature models (i.e., models consisting of demographic variables alone or demographics plus pre‐selected brain ROIs, not computed by the signature approach we describe). We used bootstrap sampling (i.e., sampling with replacement) over 10,000 iterations, generating a range of values for the differences adjusted *R*_S_^2^ − adjusted *R*_M_^2^, where *S* is the signature model and *M* another model of interest. We used the R boot package (https://cran.r-project.org/web/packages/boot/boot.pdf) in R version 3.5.1 to estimate confidence intervals for this difference at levels of 80%, 90%, 95%, and 99%. If a confidence interval was entirely above 0, we took this as evidence of better signature performance at that level of significance.

## 3 RESULTS

### 3.1 Discovery and validation data cohorts

Participant and scanner characteristics of our discovery and validation cohorts are presented in Table 1. The validation and discovery cohorts were mutually disjoint.

**TABLE 1:** Demographic profiles of the (a) discovery and (b) validation cohorts.

	ADNI 3	UCD
**(a) Discovery cohorts**		
*n*	815	576
Age, years (mean [SD])	71.4 (7.3)	76.8 (7.0)
Gender (percent female)	52	60
Education, years (mean [SD])	16.5 (2.5)	13.9 (4.1)
Race/ethnicity (percent)	Hispanic/Latino 4	Asian 3
	Not Hispanic/Latino 95	African American 23
		Hispanic/Latino 21
		White 50
		Other 2
Clinical diagnosis (percent)	CN 50	CN 56
	MCI 27	MCI 26
	AD 10 Not available 13	Demented 17
ECog Mem (mean [SD])	1.85 (0.91)	2.11 (0.96)
Neuropsych Mem (mean [SD])	0.60 (0.86)	−0.23 (0.99)
Correlation of ECog and Neuropsych Mem Domains	−0.65	−0.53
Scanner field strength (Percent 3T)	99.9	22

	ADNI 1	UCD
**(b) Validation cohorts**		
*n*	435	348
Age, years (mean [SD])	75.6 (7.1)	76.8 (7.8)
Gender (percent female)	42	62
Education, years (mean [SD])	15.5 (3.0)	13.2 (4.3)
Race/ethnicity (percent)	Hispanic/Latino 2	Asian 3
	Not Hispanic/Latino 97	African American 31
		Hispanic/Latino 24
		White 41
		Other 1
Clinical diagnosis (percent)	CN 20	CN 51
	MCI 47	MCI 28
	AD 33	Demented 21
	Not available 13	
ECog Mem (mean [SD])	Not available	2.20 (1.04)
Neuropsych Mem (mean [SD])	−0.169 (0.85)	−0.223 (1.02)
Correlation of ECog and Neuropsych Mem domains	N/A	−0.569
Scanner field strength (Percent 3T)	0	51

Abbreviations: AD, Alzheimer's disease; CN, cognitively normal; MCI, mild cognitive impairment.

In the discovery cohorts, ADNI was significantly younger (*p* < .001) than UCD. Age ranges were 55–90.6 for ADNI and 52–95 for UCD. Scatterplots of each outcome versus age revealed significant associations but relatively small cubic polynomial fit *R* ^2^ values (0.11 for UCD neuropsychological memory and less than 0.06 for all others). The best fitting cubic polynomial in each case was approximately linear for the ages between 60 and 90 (see Figure S1). This supports our inclusion of age as linear controlling variable. ADNI ECog Mem and neuropsychological memory were both significantly better than in UCD (*p* < .001 for both domains). Domain scores were significantly correlated in both cohorts (*p* < .001). The correlation is negative because higher ECog Mem scores indicate worse outcomes, while higher neuropsychological scores are better. ADNI was significantly less female (*p* < .001) and had significantly more education (*p* < .001) than UCD. ADNI was almost entirely non‐Hispanic/Latino, whereas UCD had about 50% white and almost one‐quarter each of African American and Hispanic/Latino. For clinical diagnosis, UCD had a significantly greater proportion of normal (CN) than ADNI (*p* = .008 via likelihood ratio) as well as significantly more participants with dementia (*p* < .001). In ADNI, the clinical diagnoses are principally in the Alzheimer's spectrum, whereas the UCD demented category included vascular as well as Alzheimer's disease dementias. Our last measure of scanner field strengths shows that ADNI consisted of almost entirely 3T scanners (in fact there was just one 1.5T), whereas UCD was about 78% 1.5T.

In the validation cohorts, the ages were not significantly different. ADNI was significantly less female than UCD (*p* < .001), and with significantly more education (*p* < .001). ADNI was almost entirely non‐Hispanic/Latino, whereas UCD was about 41% white. For clinical diagnosis, UCD had a significantly higher proportion of normal and significantly smaller proportions of MCI and Demented than ADNI. For field strengths, ADNI was entirely 1.5T while UCD was 51% 3T. ADNI and UCD were not significantly different for neuropsychological memory.

From each discovery cohort we selected 40 randomly chosen subsets of size 400 each, without replacement. In the ADNI discovery cohort (*N* = 815), the average pairwise intersection of subsets was about 178 participants or about 22% of the full cohort. In the UCD discovery cohort (*N* = 576), the average pairwise overlap of subsets was about 283 or about 49% of the full cohort. In each validation cohort, we selected 50 subsets of size 200. In the ADNI validation cohort (*N* = 415), the average pairwise overlap was about 92 participants or about 22% of the full cohort. In UCD validation cohort (*N* = 348), the corresponding numbers were 115 participants and 33%.

### 3.2 Replication of spatial selection: Overlap frequency maps

**FIGURE 3:**
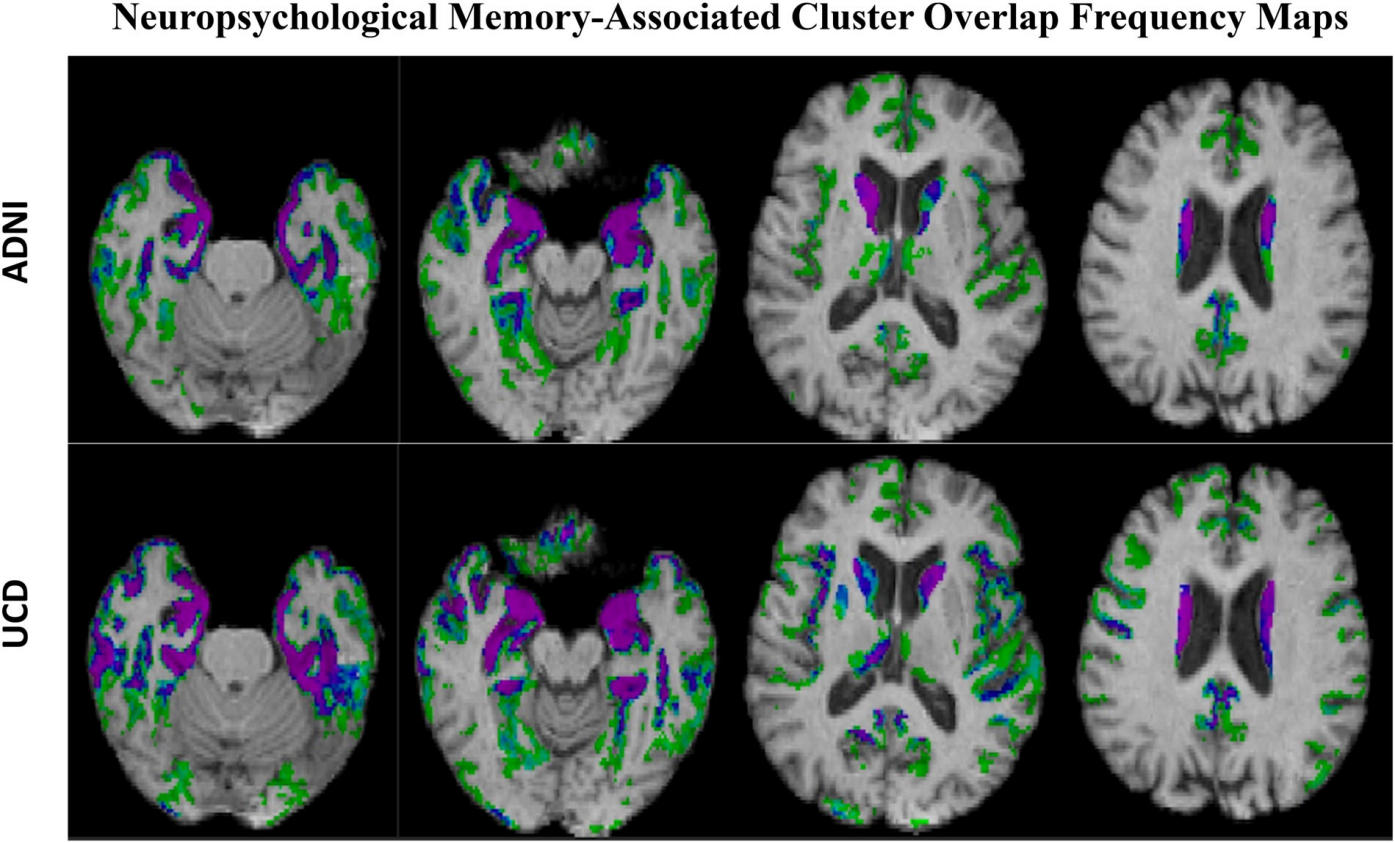
Percentage overlap of significant gray matter cluster associations at *t* = 3 to memory over 40 random trials in each cohort. Percentage frequency coding ranges from light green (2.5%, i.e., 1/40) through blue (60% or 24/40), to purple (100%, i.e., 40/40).

Figure 3 displays overlap frequency maps for cognitive memory‐association clusters in each cohort. ECog memory (not shown) exhibited similar patterns, with one exception: the caudate was not selected by UCD ECog signatures. Maps in each cohort show consensus overlaps (purple: 100%) for medial temporal, amygdala, and hippocampal locations. More dorsally, there was overlap in the caudate, though with somewhat smaller extent of the 100% regions than in the temporal slices. We also note small areas of high‐frequency overlaps in the precuneus and PCC for both ADNI and UCD (rightmost slices in each cohort).

### 3.3 Discovery cohort consensus signature models

**FIGURE 4:**
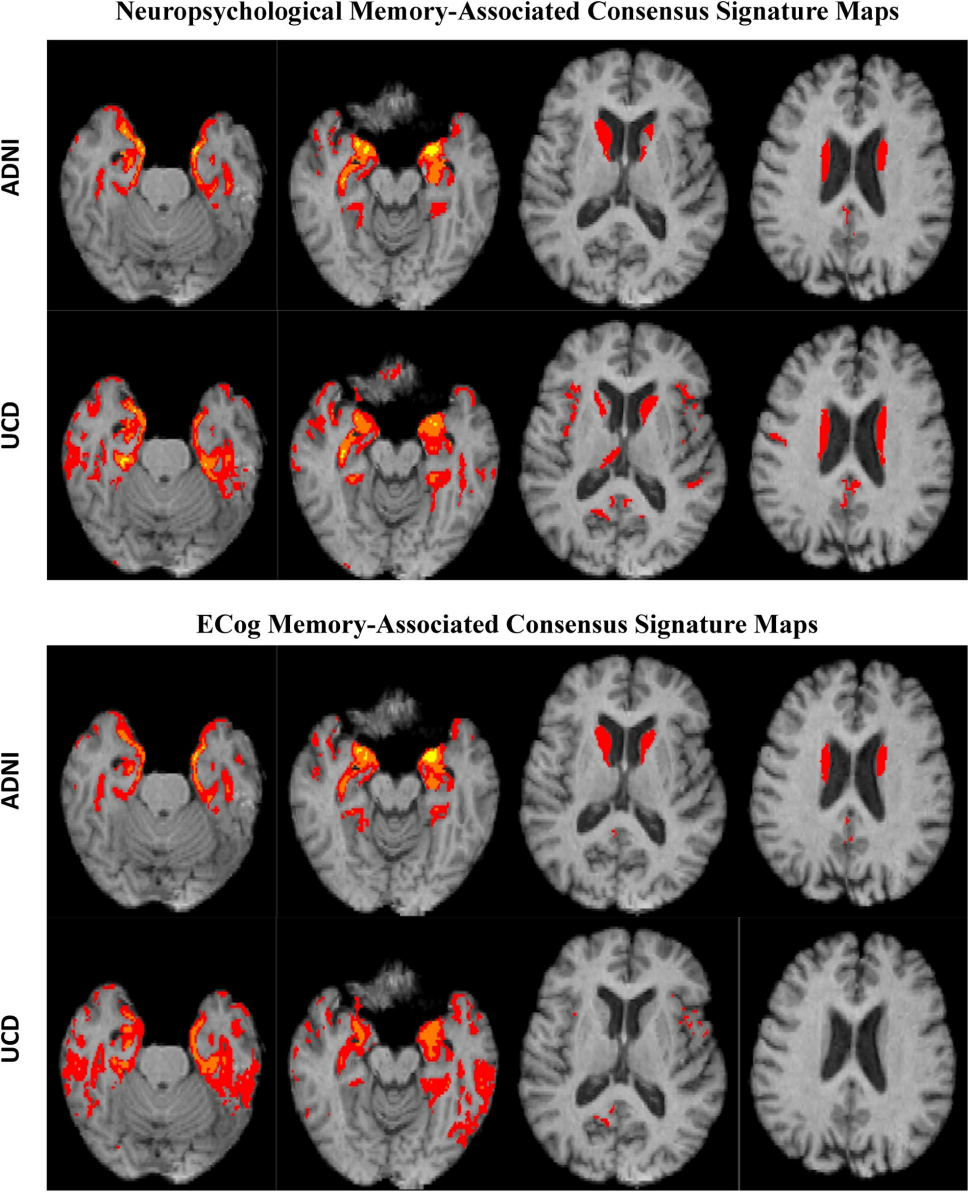
Consensus signature TsROI regions for memory (top) and ECog Mem (bottom) computed in each cohort. Based on 70% overlap “consensus” at each of three *t*‐levels of association: *t* = 3 (red), 5 (orange), and 7 (yellow).

For neuropsychological memory, consensus masks in ADNI and UCD show a reasonable convergence, each cohort having extensive associations at levels *t* = 3, 5, and 7 within temporal regions (leftmost images), and associations at *t* = 3 within the caudate (rightmost images). Each similarity metric for UCD versus ADNI (Table 2) exceeds its counterpart reported in our previous work (Fletcher, Gavett, et al., 2021). For both domain signatures in Figure 4, the UCD spatial extents appear noisier than ADNI in the *t* = 3 range, which may weaken the similarity scores. Noisiness in UCD could be due to greater pairwise overlap between the random discovery subsets, and next to 78% of the images being acquired on 1.5T scanners. In any case, our *η* ^2^ scores lie between ranges previously characterized as “reasonably similar” and “very similar” (Bakkour et al., 2013).

**TABLE 2:** Numerical pairwise similarity scores for consensus regions.

	ADNI_ECogMem	UCD Mem	UCD ECogMem
**Dice pairwise similarity scores**			
ADNI Mem	0.812	0.535	0.446
ADNI ECogMem		0.553	0.464
UCD Mem			0.582
***η* ^2^ pairwise similarity scores**			
ADNI Mem	0.926	0.804	0.756
ADNI ECogMem		0.809	0.761
UCD Mem			0.809

*Note*: Top: Dice, for single mask *t* ≥ 3. Bottom: *η* ^2^ taking into account correspondence of *t*‐value locations.

For ECog memory, the cohort consensus TsROIs are also reasonably convergent in the two cohorts, except that the UCD TsROIs do not show any association of caudate with ECog outcome. We also note that in each cohort, the signature TsROIs for neuropsychological memory and ECog memory are similar, except for the absence of caudate in the UCD ECog memory signature. In sum, consensus signature masks show decent resemblances for ADNI vs. UCD by outcome domain, and strong similarities (high Dice and *η* ^2^ scores) between outcome domains by cohort of origin.

### 3.4 Numerical similarity evaluation of discovery cohort regions of interest

Table 2 provides numerical similarity measures for the signature maps. Dice scores (Dice, 1945) measure the ratio of volumes in pairwise intersections to unions of the two masks: DICE = 2 × |*M*_1_ ∩ *M*_2_|/|*M*_1_ ∪ *M*_2_|. The maximum score is 1 when the two masks coincide. We performed pairwise Dice measurements for color‐coded regions of Figure 4 (i.e., *t* ≥ 3). *η* ^2^ is a voxelwise measure similar to cross‐correlation but preferable in this instance because it is sensitive to local and global differences in voxel‐based magnitude (Cohen et al., 2008). Unlike the Dice measure in this context, *η* ^2^ gives a summary of the degree of overlaps of individual color‐coded levels (*t* = 3, 5, 7). It takes values from 0 to 1, with 1 indicating identical images.

### 3.5 Overlaps of signature regions with brain atlas parcellations

Table 3 shows the percent overlap of selected brain atlas regions by consensus signature masks (*t* ≥ 3), in other words by all the colored regions displayed in Figure 4. All four signature maps overlapped three medial temporal structures (amygdala, entorhinal cortex, and hippocampus), at consistently high percentages of those structures (roughly 60%–95%) and except for the two UCD ECog signatures, around 45% of the caudate. The parahippocampal gyrus was overlapped at mid‐40% levels by the UCD signatures but also consistently at lower percentages by the ADNI signatures. Similar patterns are seen for the fusiform and inferior temporal regions.

**TABLE 3:** Top 15 regional atlas overlaps for consensus masks corresponding to *t* ≥ 3, sorted for the UCD memory signature overlaps.

	ADNI 3		UCD	
	ECogMem	Memory	ECogMem	Memory
Amygdala	0.94	0.93	0.91	0.98
Entorrhinal	0.72	0.74	0.78	0.77
Hippocampus	0.59	0.63	0.65	0.64
Parahippocampal	0.34	0.29	0.46	0.46
Caudate	0.47	0.43	0.03	0.44
Isthmus cingulate	0.19	0.12	0.12	0.29
Fusiform	0.14	0.13	0.31	0.26
Inferior temporal	0.10	0.07	0.49	0.22
Medial orbitofrontal	0.09	0.05	0.13	0.21
Insula	0.04	0.07	0.16	0.19
Pars orbitalis	0.0	0.0	0.06	0.18
Superior temporal	0.15	0.11	0.17	0.13
Lateral orbitofrontal	0.02	0.01	0.10	0.13
Transverse temporal	0.0	0.0	0.0	0.13
Middle temporal	0.03	0.02	0.21	0.12

*Note*: Entries show percentage overlaps of atlas anatomical regions by signatures.

### 3.6 Validation of signature model performance

Performance validation entailed the use of two additional data sets that were disjoint from the discovery sets (see Table 1—(b) Validation Cohorts). Fits were measured from adjusted *R* ^2^ of regressions including age, gender, and education as covariates (Equation 1). We first examined the comparative performance of the ADNI and UCD signature models in 50 randomly chosen subsets of size 200 within each of the ADNI and UCD validation sets. We then compared the model fits of both signature models with fits from other commonly used predictors of outcome in the full ADNI and UCD validation sets.

### 3.7 Signature performance comparisons from repeated trials in subsets of each validation set

**FIGURE 5:**
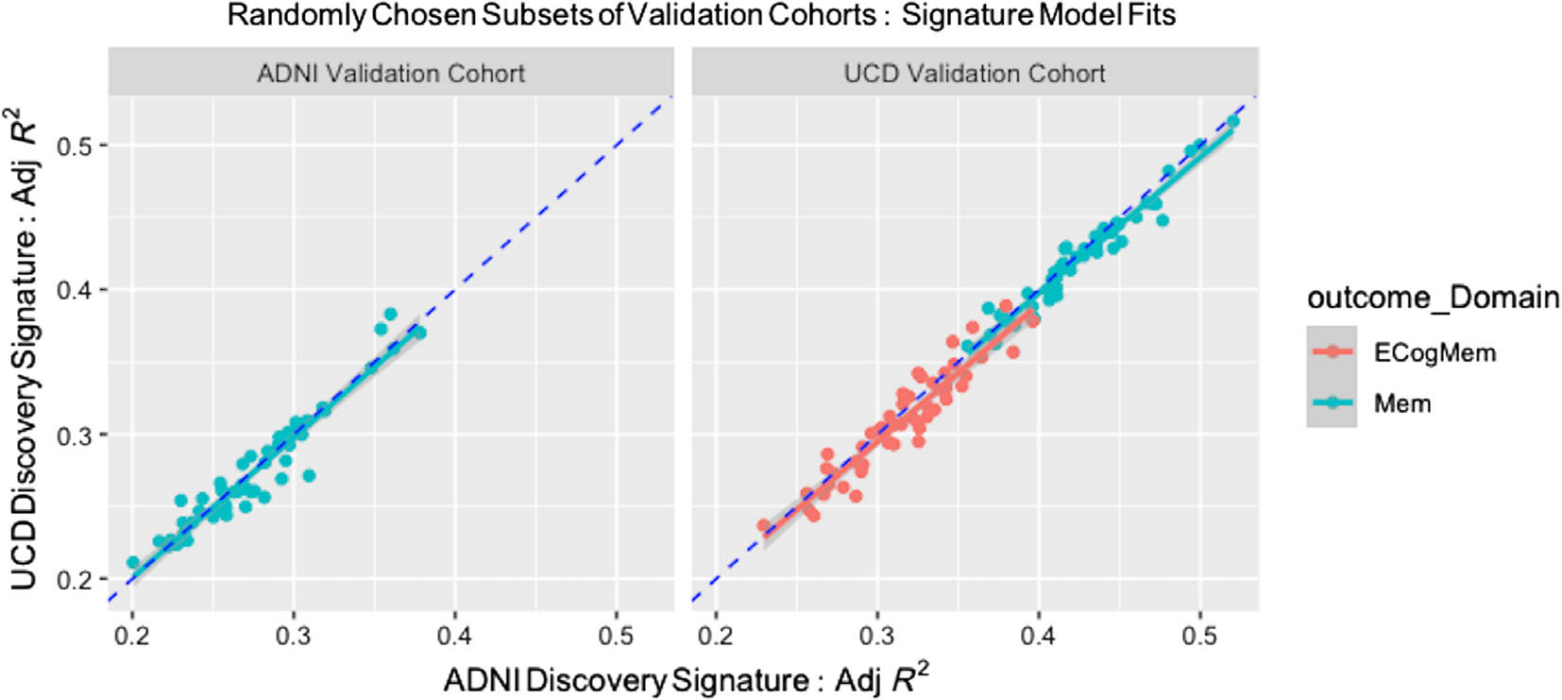
Validation of signature model fits to outcome over 50 randomly selected subsets of size 200 in each validation cohort. Validation cohorts are each disjoint from both discovery cohorts in which the signature models were computed. See the text and Table 1 for validation cohort characteristics. Models of outcome are regressions on signature models, controlling for demographics (Equation 1). Plots are the adjusted *R* ^2^ fits. The *x*‐coordinate of each point is the *R* ^2^ value for the ADNI‐signature model in a single validation subset and the *y*‐coordinate is the *R* ^2^ value for the UCD‐signature in the same subset. Thus, for example, in the “ADNI Validation Cohort” panel, the leftmost blue point indicates that the ADNI‐derived signature has an adjusted *R* ^2^ fit of about 0.21 to memory outcome, while the UCD signature has a fit of 0.22 in the same validation subset. The dashed dark blue line is the identity *y* = *x*. Blue memory points show higher outcomes in UCD than the red for ECog Memory, and higher than blue ADNI memory fits, due to demographics explaining less of the outcome variance in the latter two models (see Figure 6). Complete ECogMem values were not available for the ADNI validation cohort, so only memory outcomes are shown in ADNI validation.

Signature models generated from the ADNI and UCD discovery phases were each evaluated in every subset of each validation cohort, with model fit measured by adjusted *R* ^2^. The results are plotted in Figure 5. Scatterplot coordinates are *x* = *R* ^2^ for the ADNI‐derived signature models (*R* ^2^_ADNI_) and *y* = corresponding *R* ^2^ fit for the UCD signature models (*R* ^2^_UCD_), yielding 50 points per plot. Complete ECog memory data were not available in our ADNI 1 data consisting of older scans, so we display only results for memory in that cohort.

The scatterplots reveal tight correlations of signature model fits lying very close to the identity line (*y* = *x* with slope 1). Thus, although there is a range of *R* ^2^ values across the 50 trial subsets, fits of both signature models follow each other closely across the trials. This suggests not only strong correlation but also good agreement. A Bland–Altman analysis of the differences *R* ^2^_UCD_ − R^2^_ADNI_ versus (*R* ^2^_UCD_ + *R* ^2^_ADNI_)/2 for neuropsychological memory, using 95% confidence intervals (CI), showed a slight differential bias in favor of the ADNI‐derived signature model. In the ADNI validation set, the bias was 0.0015 but was not significant. In UCD validations, the bias was 0.004 and barely significant. Limits of agreement were slightly wider in ADNI but in both validation cohorts most differences of model fits fell within a range of the bias value ±0.02. Compared to the range of *R* ^2^ values all above 0.2, this suggests very good agreement between the signature measurements.

### 3.8 Signature performance comparisons with other models in the full validation sets

**FIGURE 6:**
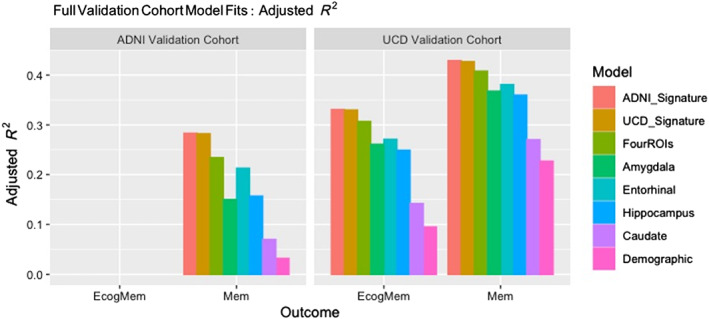
Performance comparisons over full validation cohorts. “FourROIs” designates the model incorporating amygdala, entorhinal cortex, hippocampus and caudate as multivariate predictors. “Demographic” is the model incorporating age, gender and education with no brain variables. Fits of pure demographic models are shown for comparison and vary across outcomes and cohorts. All other models incorporated the demographic variables plus brain variables as indicated. ECog memory outcome data for the ADNI validation cohort were not available.

We examined the fit performances of each cohort consensus model in each full cohort. Again, ECog memory data were incomplete for the ADNI cohort, and those results are not shown. We compared signature model performances to those of brain regions figuring prominently in the consensus models (see Table 2): entorhinal cortex, amygdala, hippocampus, and caudate, and finally a model incorporating all four of these regions as predictors (FourROIs). To demonstrate a baseline level of predicted variance from demographic factors alone, we included fits for the model incorporating age, gender, and education but no brain predictors. Results are displayed in Figure 6.

We note two main points. First, performances of the ADNI‐ and UCD‐derived signature models are almost identical in two independent validation cohorts. This is consistent with the small amount of differential bias found in the 50 repeated trials (Figure 5). Our consensus models thus may have reduced within‐discovery‐set versus out‐of‐set bias in performance, unlike the performances of signature models in our previous work (Fletcher, Gavett, et al., 2021), while also improving slightly on overall fit performance. Second, the signature models performed better than all other models tested, including the “FourROIs” model that used four atlas‐based regions most heavily overlapped by the signature regions. Also of interest, the demographic models explained less of each outcome than all other models, and their level of fit varied noticeably for different outcomes and cohorts. Statistically significant differences of the signature performance with FourROIs varied, perhaps dependent on the amount of contribution from the demographic variables. We tested this next.

### 3.9 Statistical significance of optimal performance in validation sets

To test statistical significance of signature vs. FourROIs and entorhinal model performance, we computed bootstrapped CIs for adjusted *R* ^2^ differences of these two models in the full validation cohorts. For models incorporating demographic covariates in the UCD validation cohort, both the signature neuropsychological memory models were better than FourROIs at the 80% CI level, and better than FourROIs for ECog memory at the 90% level. Meanwhile in the ADNI validation cohort, both signature models for memory were better than FourROIs at the 99% level. The next highest performing model after FourROIs was entorhinal. All signature models were significantly higher than entorhinal at the 99% level. We then tested the hypothesis that higher demographic variability in UCD (Table 1(b) Validation Cohorts) was reducing the effects of the signature models. We performed bootstrapping of other model differences in which one demographic variable was removed. Removing age or education produced significantly better signature performances at the 95% level. Removing gender gave better performance at the 99% level.

### 3.10 Interactions of signature variables with diagnosis in validation sets

We performed regressions of outcome on demographic and signature variables as in Equation (2), but now adding diagnosis (normal, mild cognitive impairment, or dementia) and its interaction with signature variables. In the UCD validation set, for each outcome of neuropsychological and everyday memory, there were significant main effects of diagnosis (*p* < .001) and each signature variable (*p* < .001) but no significant interactions. In ADNI 1, there were significant main effects of diagnosis and each signature variable (*p* < .001 in all instances) but no significant interactions in models of neuropsychological memory. ECog memory was not tested in ADNI.

### 3.11 Comparisons with signature models derived from discovery sets of different sizes

**FIGURE 7:**
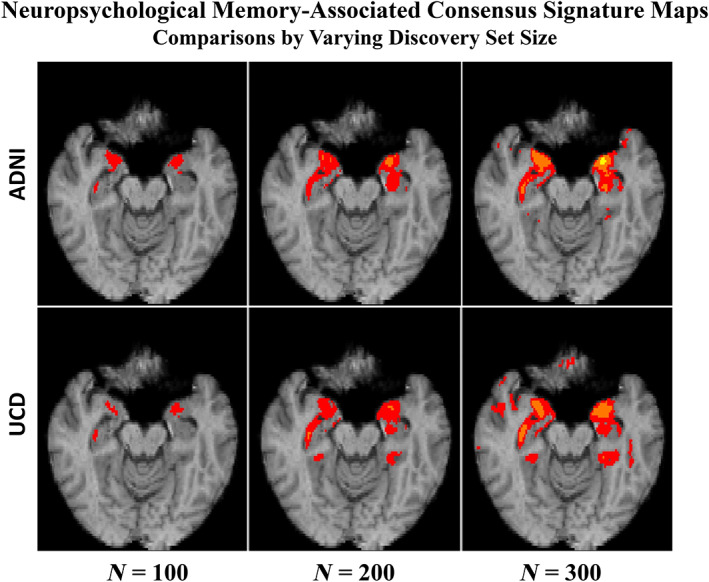
Consensus signature TsROI regions derived as in Figure 4, but using variable discovery set sizes (40 subsets at each size level = 100, 200, and 300) in each discovery cohort. Top: ADNI discovery cohort. Bottom: UCD discovery. *t*‐Levels of association: t = 3 (red), 5 (orange), and 7 (yellow). Compare these with the top two rows, second panels of Figure 4.

**FIGURE 8:**
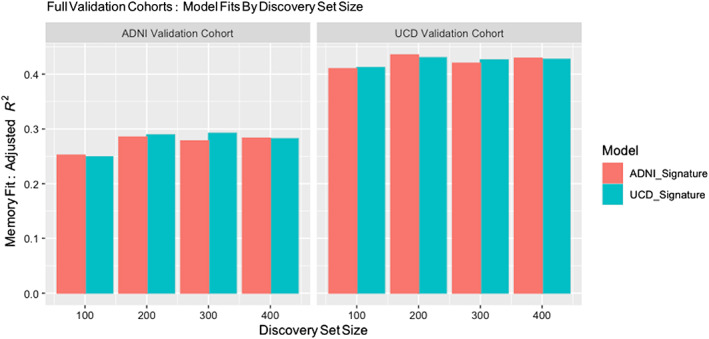
Performance comparisons for model fit of neuropsychological memory over full validation cohorts for signature models generated by discovery subsets of sizes as indicated. Compare these with the neuropsychological memory components of Figure 6.

We compared the spatial extent of signature masks and model fit performances for neuropsychological memory as outcome, using consensus models generated by 40 random discovery subsets at sizes 100, 200, and 300 in each of the ADNI and UCD discovery cohorts. Spatial extents, color‐coded by *t*‐levels of significant association, are seen in Figure 7. Plots of adjusted *R* ^2^ model performance are displayed in Figure 8.

From Figure 7, we note that spatial extents of consensus regions show up first (at discovery size *N* = 100) in regions entirely contained in the regions at larger *N*, and that these appear to expand outward with increasing *N*. Regional selection thus appears to be spatially consistent from low to high *N*, consisting largely of outward growth from already selected regions. Meanwhile, regions appearing at lower *N* values start to develop larger *t*‐values with increasing *N*. Thus, from size 300 to 400, very little new extent is added but *t*‐levels of association increase within regions already present at 300 and lower *N*. In the larger ADNI cohort we also implemented models at *N* = 500 and 600. These corroborated this pattern (data not shown).

In Figure 8, performances are uniformly high at all discovery sizes, although *N* = 100 model fits are the lowest. Interestingly, all consensus models here (even *N* = 100) outperform any competing nonsignature model (Figure 6). For other set sizes, the performances are very close to *N* = 400, and surprisingly, slightly higher than *N* = 400 in some models at size 200 or 300. At all set sizes, consensus models derived from UCD and ADNI discovery perform very similarly in both validation cohorts, suggesting minimal bias due to overlearning in the cohort where derived.

## 4 DISCUSSION

### 4.1 Summary of method and results

This project had two aims. First, we conducted a rigorous statistical validation, based on multiple tests of replicability, of the exploratory voxel‐based signature approach documented in our recent publication (Fletcher, Gavett, et al., 2021). Second, we aimed to extend the exploratory approach beyond neuropsychological memory to the outcome domain of everyday cognition (Farias et al., 2008; Farias et al., 2013), investigating similarities in brain GM substrates for these outcomes. By these, we aimed to show that reproducible brain signature phenotype generation was feasible using discovery from moderately large datasets.

### 4.2 Spatial and model fit replicability

In each of two cognitively heterogeneous discovery cohorts, 40 independent computations of ROIs associated to outcome showed high spatial replicability (Figure 3), allowing us to designate consensus regions by cohort (Figure 4). Signature models computed from cohort consensus regions achieved model fits of outcome that were highly correlated across 50 randomly chosen subsets in validation cohorts disjoint from the discovery cohorts (Figure 5). We thus leveraged spatial replicability across multiple trials to create consensus signature models having high model fit replicability, suggesting validation of these signature regions as useful brain measures. Finally, we found that these signature models achieved better explanations of outcome variance than other plausible and standardly used models (Figure 6), while also reducing in‐set vs. out‐of‐discovery‐set performance bias (Marek et al., 2022) that was present in our previous work (Fletcher, Gavett, et al., 2021).

### 4.3 Minimizing discovery set bias

A crucial issue in data‐driven approaches is the tendency to overlearn the training set, leading to inflated estimates of model performance within the training set and poorer estimates in other sets. Although the discovery and validation sets in our experiments were disjoint, they nonetheless shared educational and racial/ethnic representation of the imaging cohort from which they were drawn, and these differed between UCD and ADNI (Table 1). Racial/ethnic categories can have significant effects on cognitive outcomes, perhaps because they encompass multiple factors that are difficult to account for individually (Gavett et al., 2018). Thus, we might expect a signature model trained in one cohort to perform better than the model trained in the other cohort, when explaining outcomes within its own cohort. However, our results indicated similar performance of both signature models in both cohorts (Figure 6), suggesting that the consensus step succeeded in reducing overlearning and supporting generalizability.

### 4.4 Brain GM substrates of neuropsychological and everyday memory

Brain GM consensus regions for neuropsychological memory strongly overlap structures already known to be associated with this domain (Table 3). Structures most strongly overlapped were amygdala, entorhinal cortex, hippocampus, and caudate. These are structurally and functionally connected (Fjell et al., 2015; Fjell et al., 2016). The hippocampus and entorhinal cortex are part of a network dedicated to memory and spatial function (Rolls, 2015) and the amygdala is involved in memory‐related emotion (Catani et al., 2013; Rolls, 2015). The caudate nucleus, on the other hand, is involved in memory‐related navigation strategies alternative to those using the hippocampus (Aumont et al., 2019; Bohbot et al., 2007; Bohbot et al., 2011). Therefore, while these are not new findings of the signature approach, they do suggest a validation in established theory, supporting confidence that our approach can be used to accurately delineate brain substrates of other behavioral domains.

Application to another domain was our second aim, leading to our signature model for ECog memory. We note similarities of neuropsychological memory and ECog memory brain signatures (Figure 4 and Table 2), with one exception of missing caudate for the UCD ECog signature. This may suggest shared brain GM substrates, including medial temporal regions for both memory outcomes (Figure 4), and lesser but consistent overlaps with isthmus cingulate (Table 3). Overall signature similarities exist between both outcome domains despite the fact that neuropsychological memory (Mungas et al., 2004; Mungas, Reed, Haan, & Gonzalez, 2005) and ECogMem (Farias et al., 2008) are evaluated by different metrics (the version of ECog used here relies on third‐person informant reports). Earlier research has found correlations of ECog memory with brain measures (hippocampal and total brain volume, dorsolateral prefrontal cortex) and neuropsychological memory (Farias et al., 2013). Our findings for signature regions of ECog memory are roughly consistent with the previously found regional associations, suggesting a validation for these findings. A potentially new finding, therefore, is that neuropsychological memory and ECog memory share similar brain GM substrates, with about the same strengths of association to these substrates.

Our findings are relevant to questions of brain substrates for memory‐related cognitive decline and Alzheimer's disease. The initial stages of Alzheimer's‐related decline show brain atrophy patterns similar to normal aging but of higher magnitude, suggesting a “normalcy‐pathology homology” (Fjell et al., 2014). Early stages of AD involve atrophy in memory‐related temporal lobe structures (Fletcher et al., 2018), spatially resembling atrophy patterns of healthy normal aging that are known to accompany normal decline of memory (Fjell et al., 2013). Our signatures of brain memory substrates may therefore also be useful as signatures of incipient AD. Work is currently underway to test this concept for predicting MCI‐AD conversion.

### 4.5 Fit performance and spatial comparisons for consensus models derived from different discovery set sizes

The spatial results (Figure 7) suggest an accretion outward with increasing discovery size *N*, while regions that are present earlier develop larger *t*‐values. Thus, various *N* values do not produce fundamentally inconsistent consensus regions. The lowest size *N* = 100 selects regions that are highly associated to outcome, and those are both confirmed (by higher t values) and accreted outward with subsequent *N*. Accretion appears to slow down at higher *N*. We were unable to perform experiments using *N* > 400 in UCD, because of limits of our overall discovery cohort sizes. We hypothesize, however, that these might show a convergence to a relatively stable set of selected regions, with *t* growing stronger within existing regions, while continuing growth outward is small. This is consistent with observation that *t*‐values grow at a rate of √*N* in regions of high association between brain and output (Marek et al., 2022; Schönbrodt & Perugini, 2013), but at slower rates in regions of lower associations. The idea of spatial convergence is consistent with findings that correlations begin to stabilize at sample sizes around 250 (Schönbrodt & Perugini, 2013).

For model fit performance, we expected that the lowest fits would occur at *N* = 100. This was true, but surprisingly, those fits still outperformed other competing models (see Figure 6). More surprising was that some model fits at *N* = 200 or 300 were better than at 400 (Figure 8). We expected a monotonic increase with *N*, perhaps approaching an asymptote. We hypothesize that higher model performance (if only by a small amount) may result from discovery subsets that overlap less at *N* = 200 or 300 than at higher N in our discovery cohorts. With less overlap, there may have been more variability and less overlearning of the same features, leading to increased ability to generalize in test sets. This would suggest a limitation imposed by the size of the overall discovery cohort.

In sum, low discovery size selects consensus regions that are still highly associated to outcome, and these appear to grow outward with increasing *N*, with regions and model fit performance both tending toward a stable state. Deviations from this pattern may occur at a threshold imposed by the overall discovery cohort size, such that values of *N* beyond the threshold may result in overlearning of repeated patterns and reduced generalizability to new sets. In this analysis, drawing from imaging cohorts of much larger size could enable discovery sets of greater *N* before excessive overlap causes diminishing returns. Our method might then converge toward a more stable consensus, explaining more of the brain‐outcome association by further reducing the effects of incidental noise, while delineating more clearly the contributions of nonbrain factors.

Our model thus appears to work well with discovery cohorts of the sizes we used. It may work better with larger cohorts. It would be interesting to see results from using a very large dataset like the U.K. Biobank (Littlejohns et al., 2020; Sudlow et al., 2015), enabling discovery subsets of sizes in the thousands with very little mutual overlaps.

### 4.6 Relations to previous signature models

In the signature models of our previous study (Fletcher, Gavett, et al., 2021), cross‐validation was performed in each of three independent cohorts. In this work, we found consistency and replicability over 50 randomly selected sets in two independent validation cohorts, and over those full cohorts as well. Comparative whole‐cohort model fits displayed in Figure 6 are in line with those found in our previous work, although our current adjusted *R* ^2^ are somewhat higher overall. This suggests that the consensus step introduced here may have enhanced the model fits of our previous work, while also providing verification of replicability.

The recent empirical examination of signature replicability (Masouleh et al., 2019) suggested the multiple trials approach we followed. That work reported little replicable association between brain and behavior in a cognitively normal cohort, but found some regions selected by more than 70% of the trials for brain GM associations with short‐term memory in a cognitively mixed, clinical cohort. Reassuringly, many of their selected regions in the clinical cohort appear similar to our consensus signature TsROIs. We thus may have corroborated their results for a cognitively mixed cohort, and with even stronger associations, perhaps due to our use of larger discovery set sizes, consistent with the recommendations of that work.

### 4.7 Relations to brain atlases and theory‐driven models

High quality brain image parcellation atlases, for example, (Klein et al., 2017; Manera et al., 2020) have many benefits. They may be used directly in arbitrary study cohorts to implement “theory‐driven” models based on accumulated findings on the relationship of brain structures to behavior. They do not require computational search procedures or verification of replicability. They constitute a form of “data reduction” that is valuable for constructing tractable models. On the other hand, atlas regions do not necessarily align with networked locations that underlie behavioral outcomes of interest (Jolly & Hampshire, 2021) and this may explain why their explanatory performance is generally lower than that attained by signature models, seen in Figure 6. Recent efforts have incorporated both atlas and exploratory concepts by searching lists of atlas ROIs for a subset that optimally explains an outcome of interest (Epelbaum et al., 2018; Schwarz et al., 2016). However, using predefined atlas regions may not accurately reflect the association of ROI subregions, rather than full ROIs, with an outcome of interest. The FourROIs model (Figure 6) is a case in point. Though it incorporates the four atlas regions most heavily overlapped by our signature masks, its fit performance was still lower than the signatures. The exploratory signature approach may therefore achieve greater precision and sensitivity (i.e., selecting only relevant regions that communicate with each other for behavioral outcomes) (Genon et al., 2018; Jolly & Hampshire, 2021).

### 4.8 Strengths and limitations

An important strength of the signature approach is that it proposes hypothesis‐free, exploratory computation of brain regional measures maximally associated to outcome (Bakkour et al., 2009; Dickerson et al., 2009; Fletcher, Gavett, et al., 2021; Jolly & Hampshire, 2021). However, achieving this promise necessarily incurs conceptual and technical issues that must be addressed. First, a definitive brain signature of an outcome may not even exist, due to inter‐individual variability and the lack of repeatability even within individuals regarding brain‐behavior relations (Genon et al., 2018). Second, poor replicability and lack of association between brain and outcome, especially in healthy populations, may challenge the achievability of the signature concept (Masouleh et al., 2019). Third, behavioral outcomes depend on multifactorial arrays of brain and nonbrain factors that are difficult to fully account for (Habes et al., 2020).

These considerations suggest that there could be a ceiling for how much behavioral variance can be explained by brain models. However, the exploratory signature approach may be useful here, since rigorously derived signatures could indicate where that ceiling lies. A relevant example is cognitive reserve (CR), a construct explicitly aimed at quantifying differences between observed behavioral variance and predictions by brain models (Reed et al., 2010; Stern, 2012; Stern et al., 2018; Zahodne et al., 2013). By generating improved estimates for outcome variance explainable by brain measures (thereby putting more accurate limits on what is not CR), the signature approach could refine the quantification of CR, leading to more precise hypotheses of what other factors may be associated with it.

Given the many brain factors relevant to behavioral outcomes, an appropriate implementation of the signature approach may come from machine learning. This approach is capable of accounting for interactions between many more factors than human‐constructed models could incorporate (Dinsdale et al., 2020). Machine learning entails its own challenges, including the need for very large data sets (Fletcher, Decarli, et al., 2021) and an opacity of output that may not be readily interpretable or accessible to human understanding (Böhle et al., 2019). But it may be a feasible path toward more powerful models. Future research from our group will aim to implement this approach.

One aim of our current effort has been to address the spatial and fit replicability issues raised by (Masouleh et al., 2019). A limitation of the randomly selected subset technique is the tradeoff between lack of independence (degree of overlap) of subsets and the need for larger discovery sizes to facilitate better learning. From tests of three levels (30, 50, and 70 percent of the full cohort), Masouleh et al. found the best spatial replicability in discovery subsets of the largest size, at 70% of the full cohort or about 326 participants. In other words, larger discovery size outweighed greater overlap of discovery sets. Our ADNI discovery cohort subsets were less than 50% of the total cohort and therefore had smaller overlaps, while our UCD discovery subsets were 70% of the UCD cohort, with the same overlaps as in Masouleh et al. Thus, using discovery subsets of equal or smaller pairwise overlaps, and larger absolute size (*N* = 400 each), our results appear to be stronger than theirs, both for spatial replication of selected regions and for fit replication in separate validation sets. Nonetheless, there remain unexplained spatial differences between signatures generated in different cohorts (Figure 4 and Tables 2 and 3), and further work with larger sample sizes may help clarify this issue.

The role of demographic variables also raises issues for further work. Pure demographic models explained different amounts of outcome across outcome and cohorts (Figure 6). This probably contributed to varying signature model fits by outcome and validation set. Furthermore, demographic variables “diluted” the explanatory power of the signature variables in UCD, so that signatures were significantly better than the next best model in ADNI but not in UCD. These observations may be due to ADNI cohorts being more demographically homogeneous (Table 1), with less demographic variance to explain outcome than in UCD. Brain associations with behavioral outcomes are known to differ by racial/ethnic group (Gavett et al., 2018). Other nonbrain variables than age, education and gender may also be relevant in models of outcomes. These suggest future lines of research aimed at exploring the interactions between brain signatures and other variables.

## 5 CONCLUSION

We have conducted a refinement and rigorous validation of our previously described method, along with extending it to a second behavioral domain. First, our results support the feasibility of generating behavior‐related brain signatures that depend minimally on discovery set and can be used as robust GM brain phenotypes. Remaining differences in spatial and fit replicability suggest further investigation with larger datasets, to explore cohort‐based differences in signature models and develop signatures incorporating multiple brain measures beyond GM. Second, we found that GM brain substrates for neuropsychological and everyday function memory are convergent. This is a new finding that warrants further exploration.

## FUNDING INFORMATION

This project was funded by R01 AG052132 (NIH/NIA).

## CONFLICT OF INTEREST STATEMENT

The authors declare no conflicts of interest.

## Supporting information


**Figure S1A.** Plots of neuropsychological memory vs. age in discovery cohorts (ADNI left, UCD right).

**Figure S1B.** Plots of ECog Memory vs. age in discovery cohorts.

## Data Availability

The data that support the findings of this study are available from the corresponding author upon reasonable request.
