# Supplementary figures and images for: Toward a statistical validation of brain signatures as robust measures of behavioral substrates
Source: Hum Brain Mapp. 2023 Mar 20;44(8):3094–111. doi: 10.1002/hbm.26265 (PMC10171525; doi:10.1002/hbm.26265)

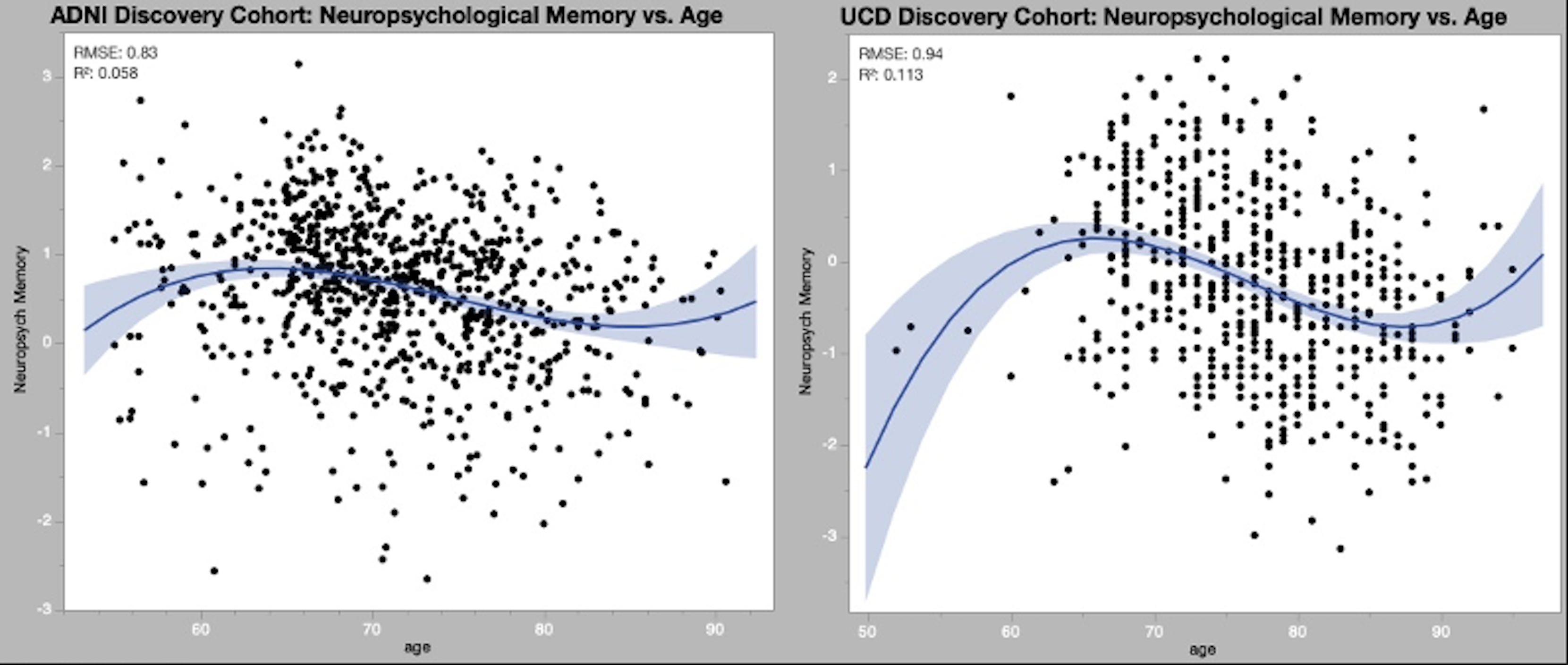

Supplement: Supplementary file 1 — Figure S1A. Plots of neuropsychological memory vs. age in discovery cohorts (ADNI left, UCD right). [file HBM-44-3094-s002.png]

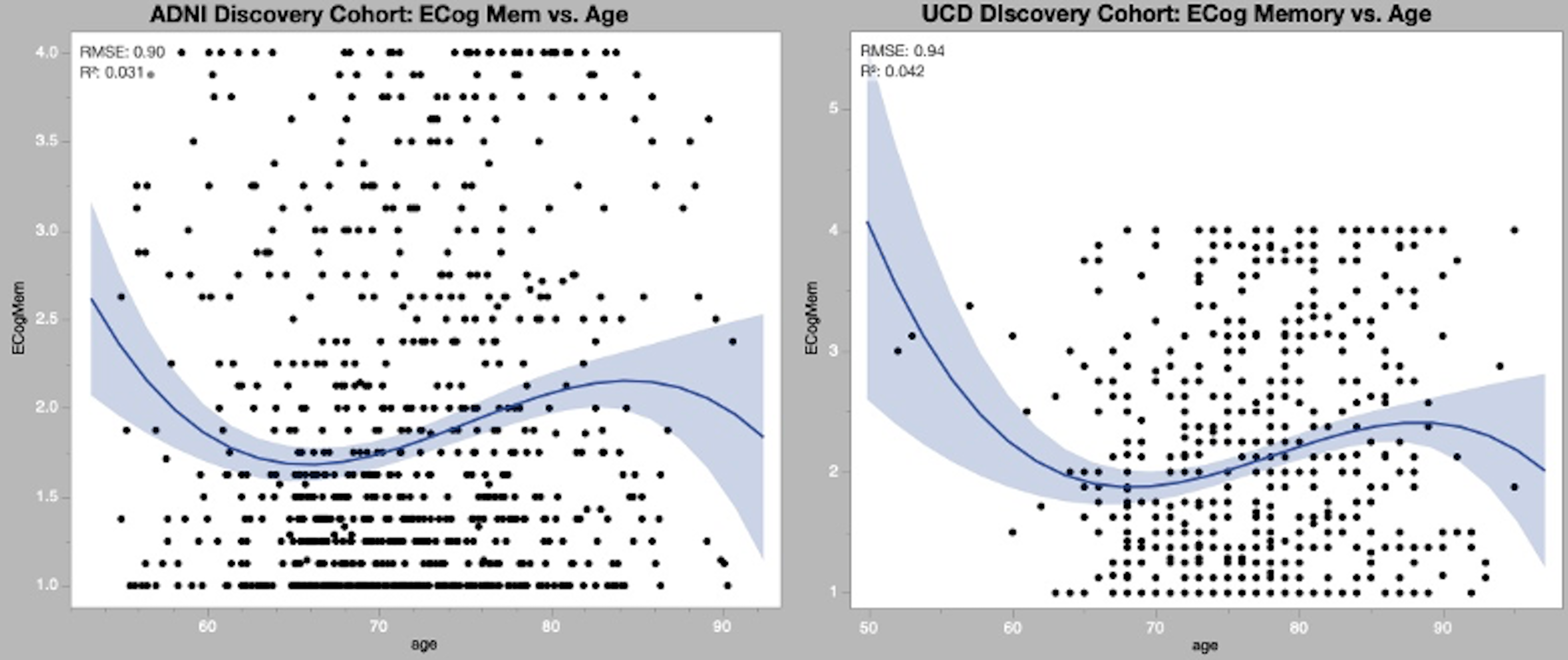

Supplement: Supplementary file 2 — Figure S1B. Plots of ECog Memory vs. age in discovery cohorts. [file HBM-44-3094-s001.png]
